# Climate effects on the diversification strategy of export firms: Evidence from China

**DOI:** 10.1371/journal.pone.0308391

**Published:** 2024-08-16

**Authors:** Junmei Zhang, Lianying Yao

**Affiliations:** 1 Global Institute for Zhejiang Merchants Development, Zhejiang University of Technology, Hangzhou, Zhejiang, China; 2 Institute for Industrial System Modernization, Zhejiang University of Technology, Hangzhou, Zhejiang, China; Shandong University of Science and Technology, CHINA

## Abstract

Using a multi-product trade model, this study investigates the impact of temperature on firms’ diversification in the export market. Using export and meteorological data of Chinese firms from 2000 to 2016, the empirical results confirm an inverted U-shaped relationship, implying that extreme temperatures significantly reduce firms’ export product diversification. The analysis shows that extreme temperatures primarily reduce the variety of both new and existing products, with a less robust effect on product exit strategies. General trade firms are more adaptable to extreme temperatures than processing trade firms, and are likely to maintain diversified strategies. Stronger regional financial markets and higher energy consumption increase the adaptability of local firms to extreme temperatures. Firms have not yet adapted to local climatic norms. Furthermore, extreme temperatures also partly inhibit diversification of export destinations and relationships. The results of the study show that as climate change intensifies, leading to more frequent extreme temperatures, firms will face significant hurdles in pursuing diversified development strategies, pointing to increasing challenges ahead.

## Introduction

As climate change accelerates, extreme temperatures are becoming more common around the world. In 2023, Europe experienced its warmest winter on record, while the northeastern United States experienced a severe cold snap. Summer heatwaves swept across densely populated countries such as the United States, China, and India. The World Meteorological Organization’s 2023 Global Climate Status Report declared it the hottest recorded year on record. Extreme temperatures have widespread economic impacts, causing labor and capital outflows due to adverse working conditions [1–3], and hindering human capital accumulation through reduced productivity and health issues [4,5]. Together, these factors erode regional economic competitiveness by disrupting local industries and deterring new investments. Mitigating the physical risks of climate change is an increasingly urgent global challenge.

The prevailing response to climate change is centered on ’mitigation’ and ’adaptation’. Academic research emphasizes reducing emissions to mitigate climate warming, yet the analysis of economic risks and resilience among various stakeholders in the face of warming remains relatively poor. This gap in research constrains the formulation of effective adaptation strategies. This study addresses this deficiency, concentrating on the adaptation of Chinese export firms to the escalating frequency of extreme temperatures attributed to climate change, and discerning which firm types are more susceptible. Specifically, we examine the sensitivity of these firms’ diversification strategies to extreme temperature. Our findings aim to provide policy insights by elucidating the influence of extreme temperatures on the diversification strategies of export firms in the world’s foremost goods trading entity.

Diversification is an important way for firms to expand their market presence [6]. Upon analyzing China’s export data from 2000 to 2016, we found that more than 80% of firms exported more than one product, which together accounted for 94% of total export value. Diversified firms show resilience in the market by maintaining the flexibility to expand in favorable conditions and by mitigating risk through diversified investments [7]. Conversely, in adverse conditions, firms can maintain profitability by strategically focusing resources on their most competitive core products [8,9]. Dynamic internal resource allocation, achieved by introducing new products and discontinuing less profitable ones, enables effective responses to market fluctuations and unforeseen challenges.

In the study, we integrate the temperature variable into a multi-product firm trade model. Through theoretical analysis, we postulate a fundamental hypothesis of an inverted U-shaped relationship between temperature and the export product diversification of firms. Underpinned by this hypothesis, we extend our methodology to employ a non-parametric econometric model. We leverage comprehensive Chinese customs export data, complemented by daily meteorological data. The principal findings of our empirical inquiry are delineated as follows:

First, temperature has a significant inverted U-shaped relationship with export product diversification, with both positive and negative deviations from the optimal temperature reducing the diversity of exported products. The more extreme the temperature, the stronger the effect. For temperatures below -2°C, each additional day results in a decrease of 0.22 in the number of exported product categories. Likewise, for each day with an average temperature exceeding 27°C, there is a decrease of 0.09 in the number of exported product categories.

Second, processing trade firms show lower adaptability to extreme temperatures than general trade firms, resulting in a greater reduction in the diversity of their export products. A mature financial market is conducive to improving the ability of local firms to adapt to extreme temperature variations, but an increase in the level of economic development does not seem to have the same effect. Firms located in developed cities tend to reduce the diversity of export products under extreme temperatures. However, increased energy consumption can mitigate this negative impact. Finally, firms have not adapted to the local temperature norms, as those in hotter regions still experience greater negative impacts from high-temperature shocks.

Third, temperature shocks primarily affect the diversification of exported products by influencing the entry and survival dynamics of products. Specifically, extreme temperatures lead to a significant reduction in the number of new and existing product categories. Finally, further examination of the influence of temperature reveals an inverted U-shaped relationship with the diversification of both export destinations and relationships (i.e. country-product pairs). However, the effects are asymmetric; low temperatures more robustly impact the diversification of export market destinations, while high temperatures more significantly affect export market relationships.

The existing literature relate to our topic primarily investigates two aspects. Firstly, research on the determinants of firms’ diversification strategies. At the theoretical level, Melitz (2003) [10] introduces a foundational model for homogeneous single-product firms, which Bernard et al. (2010) [11] subsequently extend to encompass multi-product firms. Their research indicates that firms adjust their diversification levels in response to various exogenous shocks, including technological advancements and fluctuations in international trade. Additionally, empirical studies explore how market environment changes influence firms’ diversification decisions, considering factors such as trade environment improvements [12–14], financial market development [15–17], and shifts in factor markets [18]. However, current research overlooks the impact of the physical environment, such as temperature, on firms’ export diversification decisions.

Secondly, research on the economic impacts of temperature shocks. This body of research can be broadly divided into two areas of focus. The first area examines macroeconomic impacts, with some scholars arguing that temperature shocks significantly affect only poor regions [19,20], while others contend that such shocks reduce economic performance across both developed and developing countries [21–23]. The second area focuses on micro-level firm studies, using data from various countries to explore the effects of temperature shocks on firm output [24], profit [25,26], productivity [27–29], employment [30], exports [31,32], and product quality [33]. However, these studies have overlooked firms’ diversification behavior. Given the critical impact of diversification on firms’ internal resource allocation and international competitiveness, a systematic investigation of this area is essential.

This study makes distinct contributions to the existing literature. Theoretically, we innovatively incorporate temperature factors into the multi-product trade theory model framework of Bernard et al. (2010) [11], deducing a mathematical relationship that indicates an inverted U-shaped relationship between temperature and the diversification of a firm’s export products. Empirically, our study belongs to the realm of micro-level research. Unlike existing literature that primarily focuses on firm-level performance, we are more concerned with whether temperature shocks will alter a firm’s export diversification strategy, specifically if firms will respond by changing the number of export products and export relationships. To investigate this, we use data from Chinese exporting firms. Additionally, we reveal the effect of temperature on intra-firm resource allocation from the perspective of the entry and exit of export products. Finally, we explore the climate adaptability of firms from the perspectives of export modes, regional financial development, economic development, energy usage, and the geographical location of firms.

The subsequent sections of the paper are organized as follows. The Theoretical framework section outlines the theoretical model and provides the conceptual framework for our analysis. The Empirical methodology and data description section details the empirical methodology and provides a comprehensive overview of the data used. The Empirical results section presents the baseline regression and sensitivity analysis. The Climate adaptation section investigates the climate adaptability of firms. The Structural factors in export diversification decline section examines the structural factors influencing export product diversification under extreme temperature. The Extensive research section examines the impact of temperature on the diversification of firms’ export destinations and relationships. The Discussion section addresses the contributions of this paper to the existing literature, its limitations, and potential directions for future research. The Conclusion section summarizes the research findings and further proposing policy recommendations.

## Theoretical framework

This study builds on the multi-product firm trade framework of Bernard et al. (2010) [11]. By incorporating temperature elements, it formulates a theoretical model to explore how temperature shocks affect firms’ decisions to diversify their exported products. The specific model is presented below.

### Consumer side

Assuming a representative consumer in a country with a utility function characterized by a constant elasticity of substitution (*σ*>1), the function is expressed as:

$$U={\left\{{\int_{\omega \in \Omega }\left[q(\omega )\right]}^{\frac{\sigma -1}{\sigma }}d\omega )\right\}}^{\frac{\sigma }{\sigma -1}}$$ (1)

Here, *ω* represent a specific product category, Ω is the set of all product categories exported, and *q*(*ω*) denotes the quantity of product *ω* exported.

The demand function is derived by maximizing consumer utility, as shown in the subsequent equation.


$$q(\omega )=p_{\omega }^{-\sigma }P^{\sigma -1}Y$$ (2)

Where, *p*_*ω*_ is the export price of product *ω*, *P* is the price index, $P={\left(\int_{\omega \in \Omega }p_{\omega }^{1-\sigma }d\omega \right)}^{\frac{1}{1-\sigma }}$, and *Y* is the national income.

### Production side

Assuming a Cobb-Douglas production function, firms produce product *ω* using both labor (*l*) and capital (*k*). The production function is expressed as follows:

$$q_{\omega }(T)=\varphi_{\omega }(T)l^{a}k^{b}$$ (3)

Where, *φ*_*ω*_ is productivity, *T* is temperature, *a* and *b* are the proportions of labor and capital inputs, satisfying *a*+*b* = 1.

Productivity is modelled following Chatterjee et al. (2013) [16] as:

$$\varphi_{\omega }(T)=\varphi (T)\eta_{\varphi }^{-(n-1)},\;\eta_{\varphi }>1$$ (4)

Where, *η*_*φ*_>1 represents product gradient. The parameter *n* indicates the ranking of the distance of product *ω* from the firm’s core competitive product (i.e. the number of product categories). if *n* = 1, representing the core product, *φ*_*ω*_ decreases as *n* increases, indicating a greater distance from the firm’s core product. For the firm’s productivity, existing research shows an inverted U-shaped relationship with temperature, revealing an optimal temperature T* at which productivity peaks [24,27]. Specifically, for *T*<T*. *φ*′(*T*)>0, and for *T*>T*, *φ*′(*T*)<0.

### Optimal export decision

Assuming a fixed export cost *f*_*e*_ for product *ω*, the profit function for the exported product is expressed as:

$$\Pi_{\omega }=(p_{\omega }-MC)\times q_{\omega }-f_{e}$$ (5)

Where *MC* is the marginal cost of producing product *ω*. The marginal cost is derived from cost minimization:

$$MC=[{\left(\frac{\alpha p_{k}}{b}\right)}^{b}+{\left(\frac{b}{\alpha p_{k}}\right)}^{b}\times p_{k}]\times \frac{1}{\varphi_{\omega }}$$ (6)

Where labor wages are normalized to 1, *p*_*k*_ denotes the price of capital. Applying the profit maximization condition and combining Formulas (2) and (5), we derive the following expression:

$$p_{\omega }=\frac{\sigma }{\sigma -1}\times MC$$ (7)

This leads to the optimal profit:

$$\Pi_{\omega }^{*}={\left[\frac{P\times \varphi_{\omega }\times (\sigma -1)}{A{p_{k}}^{b}\sigma }\right]}^{\sigma -1}\times \frac{Y}{\sigma }-f_{e}$$ (8)

Where $A={\left(\frac{a}{b}\right)}^{b}+{\left(\frac{b}{a}\right)}^{a}$.

When a firm adds products, it increases its sunk costs and reduces its profits [34]. Therefore, if a firm produces *n* products, it is expected that product n* should satisfy $\Pi_{\omega }^{*}=0$. If this condition is not met, the firm would continue to produce new products. Combining Eq (4) with the zero-profit condition, we obtain the following equation:

$$\eta_{\varphi }^{1-n*}=\frac{1}{\varphi (T)}\times \frac{A{p_{k}}^{b}}{(\sigma -1)P}\times {\left(\frac{\sigma }{Y}\right)}^{\frac{\sigma }{\sigma -1}}{f_{e}}^{\frac{1}{\sigma -1}}$$ (9)

### Extreme temperatures and export product diversification

According to Eq (9), the derivative of the number of exported product categories (n*) with respect to temperature gives the following equation:

$$\frac{\partial n^{*}}{\partial T}=\frac{\varphi^{\prime }(T)}{\varphi (T)\times \ln\eta_{\varphi }}$$ (10)

As *φ*(*T*) and ln*η*_*φ*_ are both positive, the sign of n*′(*T*) depends on the *φ*′(*T*). Thus, for *T*<T*, indicating temperatures below the optimum, the number of exported product categories n* increases with increasing temperatures, promoting diversification. Conversely, for *T*>T*, temperatures above the optimum lead to n*′(*T*)<0. In this scenario, increasing temperatures lead to a decrease in the number of exported product categories, reducing diversification. In summary, export diversification has an inverted U-shaped relationship with temperature. Extreme temperatures have an unfavorable effect on the implementation of diversification strategies by firms.

## Empirical methodology and data description

### Empirical methodology

Following Deschênes and Greenstone (2007) [35], this study uses a non-parametric model to analyze the impact of daily extreme temperature increases on firms’ export diversification strategies. The use of this model has several advantages: Firstly, the non-parametric model’s flexibility in specification enables the examination of the non-linear relationship between temperature and export diversification without necessitating the pre-specification of a functional form. Secondly, the model includes a series of temperature bin variables with interval lengths of 5°C. These variables cover all temperature bins, ranging from the number of days with temperatures below -7°C, the number of days between -7°C and -2°C, and so forth, up to the number of days with temperatures above 28°C. This illustrates the distribution of daily temperatures over a year. For instance, if the temperature on a particular day is 0°C, that day will be counted in the -2°C to 3°C temperature bin. This methodology facilitates the direct observation of the impacts of extreme temperatures, both high and low. In contrast, when using a parametric model, the annual average temperature can mask the effects of daily extreme temperatures, significantly reducing the information contained in the estimation results. In the following sections, we conduct robustness tests using a parametric regression model. Specifically, we construct the non-parametric model as follows.


$$Diversity_{icrt}=\alpha +\sum_{m\neq 5}\beta_{1}^{m}Tbin_{crt}^{m}+\delta_{1}X_{crt}+\gamma_{i}+\gamma_{rt}+\varepsilon_{icrt}$$ (11)

The indices *i*, *c*, *r*, and *t* represent firms, cities, provinces, and years, respectively. The dependent variable, *Diversity*_*icrt*_, indicates the diversity of products exported by firm *i*, measured by the number of product categories exported by a firm. The core explanatory variables, $Tbin_{ct}^{m}$, denote the number of days in city *c* in year *t* with a daily average temperature within the temperature bin *m*. Temperature bin variables are defined with a length of 5°C, for a total of 9 bins. Where, *Tbin1 ct* represents days with a daily average temperature below -7°C. For example, if a city experiences temperatures below -7°C for 10 days in a year, then the value of *Tbin1 ct* is 10. *Tbin2 ct* represents days with a temperature in the bin [-7, -2)°C. *Tbin3 ct* represents days with a temperature in the bin [-2, 3)°C, and so on, with the final bin, *Tbin9 ct*, indicating days with a daily average temperature ≥28°C. The regression uses the temperature bin (8, 13°C (*m* = 5) as the base group. Given that the annual total of days is fixed, the cumulative days across all temperature bins remains constant. To mitigate multicollinearity in the regression analysis, one temperature bin must be designated as the baseline, thereby excluding it from the set of variables. This baseline selection is critical as it should ensure that the coefficients associated with all other temperature bins are negative, thereby signifying it as the optimal temperature range [4,24]. In accordance with this criterion, this study selects the temperature range of 8, 13°C as the baseline. The coefficient *βm 1* is of primary interest, representing the change in product diversity for each additional day with a daily mean temperature in the *m*th bin relative to the baseline. We expect *βm 1* to be consistently negative, reflecting an inverted U-shaped trend based on our theoretical model.

Control variables, denoted by *X*, include city-level and temperature-related factors, such as precipitation (*Prec*), represented by the annual mean precipitation in the city, wind speed (*Wind*), represented by the annual mean wind speed in the city, sunshine duration (*Sun*), represented by the annual mean sunshine duration in the city, humidity (*Wet*), represented by the annual mean relative humidity in the city, and atmospheric pressure (*Atmo*), represented by the annual mean atmospheric pressure in the city. To reduce omitted variable bias, we include firm (*γ*_*i*_) and province-year (*γ*_*rt*_) fixed effects in the model. *ε* represents the error term. Standard errors are two-way clustered at the firm and city-year levels [36].

### Data description

This section outlines the use of two key datasets: meteorological data and firm export data. The meteorological data, obtained from the National Meteorological Information Center, covers daily observations from 1 January 1951 to the present, including variables such as daily average temperature, maximum and minimum temperatures, 20-20-hours precipitation, average relative humidity, and average wind speed. The article focuses on data from 833 continuously operating meteorological stations between the years 2000 and 2016. Using a nearest-neighbor approach based on station coordinates and county-level centers, each county is matched with its closest meteorological station. City-level meteorological data are derived by averaging data from all stations within the same city.

Firm export data are mainly taken from the China Customs database (2000–2016). It contains import/export information at firm-product-destination-year level. The product codes in the customs database have undergone changes in various years, such as 2002, 2007, and 2011. We have standardized the codes to the HS2002 version and defined product categories based on the first six digits of the HS2002 version. For the purposes of this study, only export data is kept and the data are aggregated at the firm-year level. Therefore, we can obtain detailed firm export behavior data at the firm-city-year level, including the number of export product categories, the number of export destinations, and the number of export relationships for each firm. The China Customs database reports the cities where exporting firms are located, and we have also obtained city-level meteorological data. Therefore, we match the two databases using city codes.

Table 1 presents descriptive statistics for the variables used in this study. Firms show considerable diversity in the number of product categories exported. Some firms export only one product, while others export up to 4285 product categories at the 6-digit HS2002 level. The climate data also show considerable variation between China’s cities. For instance, some cities have average daily temperatures below -7°C for more than 149 days a year, while others have temperatures consistently above -7°C. Similarly, in terms of high temperatures, some cities have 163 days a year when temperatures do not fall below 28°C, while others have average daily temperatures below 28°C. These variations in both export strategies and climatic conditions across different firm locations provide a robust empirical basis for our research.

**Table 1:** Summary statistics.

Variables	Obs	Mean	SD	Min	Max
Diversity	3,149,535	14.43	49.97	1	4285
<-7°C	3,126,982	4.872	16.61	0	149.4
-7~-2°C	3,126,982	8.739	14.89	0	93.82
-2~3°C	3,126,982	22.41	20.40	0	99.37
3~8°C	3,126,982	36.86	22.17	0	98.67
13~18°C	3,126,982	43.44	15.26	0	113.33
13~18°C	3,126,982	52.76	12.41	0	154.2
18~23°C	3,126,982	70.25	13.05	0	181.7
23~28°C	3,126,982	81.91	27.29	0	224
≥28°C	3,126,982	44.04	27.33	0	163
Prec	3,126,982	3.452	1.504	0.0529	8.594
Wind	3,126,982	2.427	0.548	0.546	5.692
Sun	3,126,982	5.268	0.924	2.017	9.694
Wet	3,126,982	4.261	0.0972	3.322	4.515
Atmo	3,126,982	100.4	2.553	59.24	101.7

## Empirical results

### Baseline regression

**Fig 1:**
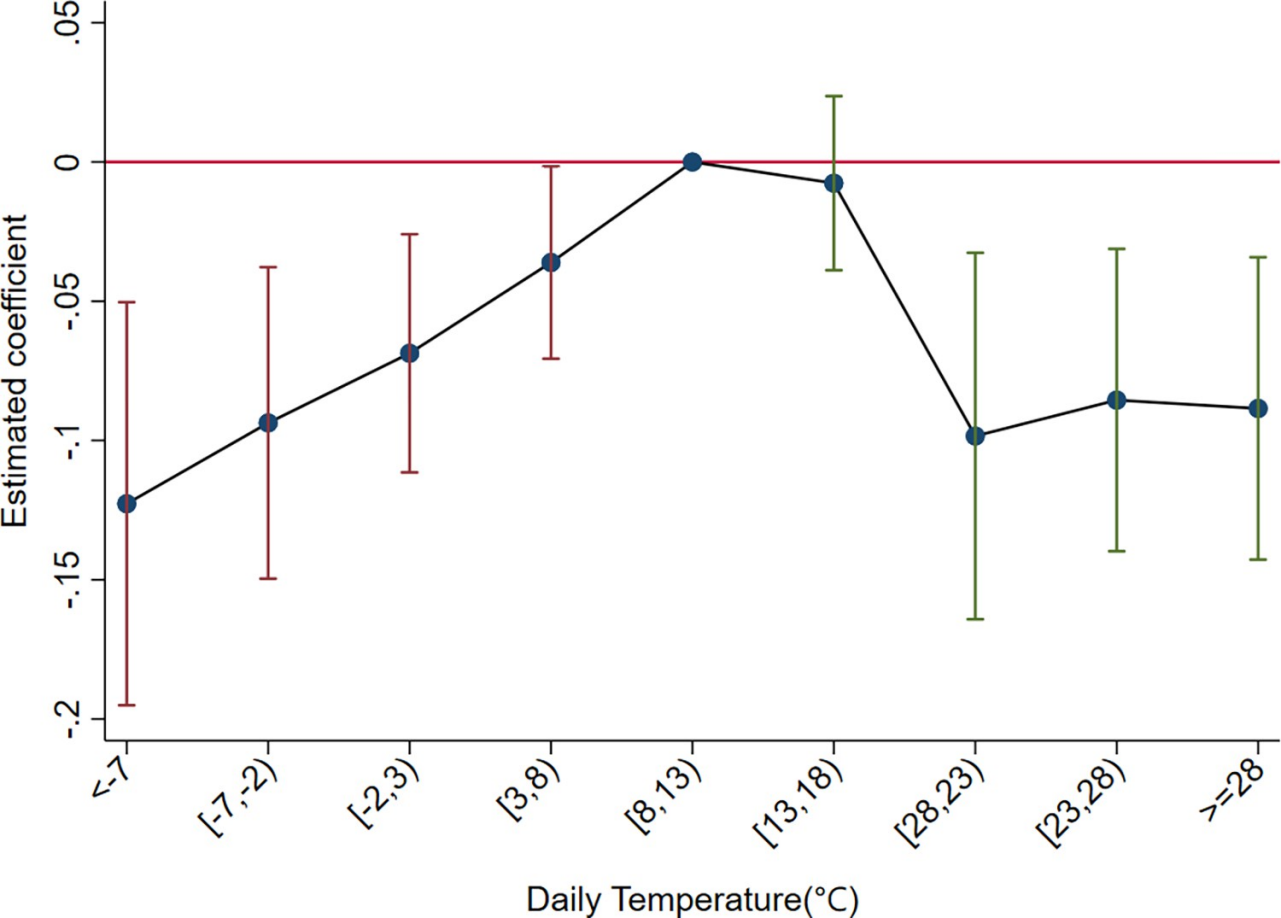
The U-shaped relationship between temperature and the product diversification of export firms. The graph is created based on the regression coefficients and standard deviation data for each temperature bin from column (3) of Table 2.

We examine the relationship between temperature and firms’ export product diversity using Eq (11). Table 2 shows the baseline regression results. In column (1), without controlling for additional variables and fixed effects, a U-shaped relationship between temperature and export diversity is observed, but with a modest R^2^ of 0.11, indicating the presence of an important uncontrolled effect. Introducing firm and province-year fixed effects in column (2) reveals predominantly negative and significant coefficients for temperature bins. The R^2^ increases to 0.737, highlighting the need to control for the fixed effects in Eq (11). In column (3), when other meteorological variables that are highly correlated with temperature are included in the model, all temperature bins have negative and statistically significant coefficients, showing an inverted U-shaped relationship (see Fig 1). Firms tend to reduce export diversity as temperature deviates from the optimum, and the reduction intensifies with larger deviations. The above results are consistent with the theoretical model estimates.

**Table 2:** Baseline regression results.

Variables	(1)	(2)	(3)
	Diversity	Diversity	Diversity
<-7°C	0.1976***	-0.1196***	-0.1227***
	(0.003)	(0.044)	(0.044)
-7~-2°C	0.0571***	-0.0855***	-0.0937***
	(0.004)	(0.033)	(0.034)
-2~3°C	-0.0076**	-0.0682**	-0.0687***
	(0.003)	(0.027)	(0.026)
3~8°C	0.0477***	-0.0332	-0.0361*
	(0.004)	(0.022)	(0.021)
13~18°C	-0.0091**	0.0102	-0.0076
	(0.004)	(0.021)	(0.019)
18~23°C	0.2002***	-0.0827**	-0.0984**
	(0.003)	(0.040)	(0.040)
23~28°C	0.0758***	-0.0600*	-0.0855**
	(0.003)	(0.032)	(0.033)
≥28°C	0.1702***	-0.0374	-0.0885***
	(0.003)	(0.029)	(0.033)
Prec			0.0089
			(0.283)
Wind			-0.9089
			(0.641)
Sun			-0.5178
			(0.373)
Wet			2.1966
			(3.656)
Atmo			6.9357***
			(1.781)
Constant	-15.8563***	30.3267***	-663.8836***
	(0.855)	(7.395)	(174.911)
Firm FE	No	Yes	Yes
Province×Year FE	No	Yes	Yes
Observations	3,125,406	3,015,824	3,015,824
R^2^	0.011	0.737	0.737

Note:

*, **, *** represent statistical significance levels of 10%, 5%, and 1%, respectively. The parentheses in columns (2)-(3) contain standard errors clustered at the firm and city-year levels. In the subsequent text, unless otherwise specified, the parentheses will indicate standard errors clustered in two-way style.

In terms of economic significance, each additional day below -2°C reduces the number of product categories exported by firms by 0.22 (0.1227+0.0937). On average, Firms face 13.6 days below -2°C per year, resulting in a reduction of 2.94 categories. Similarly, each additional day ≥28°C reduces the number of exported categories by 0.09. Firms experience an average of 44 days ≥28°C per year, resulting in a reduction of 4 categories. Extreme temperature shocks due to climate change warrant increased attention from an economic perspective for firms and nations.

### Sensitivity analysis

#### Endogeneity issue

This section considers the potential problem of omitted variables, selection bias, and bidirectional causality in the model specification. Firstly, we discuss the potential problem of omitted variables. In the baseline regression, we follow the common practice in the existing literature by controlling for other meteorological variables that are highly correlated with temperature in the model [27]. We also include province-year fixed effects to control for regional economic development levels and policy effects, and firm fixed effects to control for time-invariant firm characteristics. The above model is designed to mitigate the issue of omitted variable problem. To further validate the robustness of the conclusions, we extend the model (11) by adding city-year level economic variables, including: economic development level (*Eco*), represented by the logarithm of the city’s GDP; population size (*Pop*), represented by the total population at the end of the year; green innovation capacity (*Envinno*), represented by the logarithm of the number of green invention patents filed in the city in the current year; city openness (*Open*), represented by the logarithm of the actual foreign investment in the city in the current year; and local government capacity (*Gov*), represented by the share of local fiscal budget expenditure in local GDP. These additional control variables cover aspects such as market, government, factor endowment, and technological development, providing a comprehensive control for the economic development trends at the city-year level. The regression result with these additional variables is shown in column (1) of Table 3. The result shows that the addition of these control variables does not change the conclusions of the baseline regression, indicating that the baseline model has good explanatory power.

**Table 3:** Endogeneity issue.

	(1)	(2)	(3)
Variables	Diversity	Diversity	Diversity
<-7°C	-0.1453***	-0.1181***	-0.1061*
	(0.047)	(0.044)	(0.056)
-7~2°C	-0.1001***	-0.0891***	-0.1003**
	(0.035)	(0.033)	(0.047)
-2~3°C	-0.0704***	-0.0641**	-0.0766**
	(0.027)	(0.026)	(0.034)
3~8°C	-0.0319	-0.0317	-0.0407
	(0.021)	(0.020)	(0.029)
13~18°C	0.0015	-0.0087	0.0064
	(0.019)	(0.019)	(0.026)
18~23°C	-0.1020**	-0.1057***	-0.0914**
	(0.040)	(0.040)	(0.046)
23~28°C	-0.0934***	-0.0962***	-0.0835**
	(0.033)	(0.034)	(0.041)
≥28°C	-0.0920***	-0.1057***	-0.0876**
	(0.033)	(0.035)	(0.039)
CV	Yes	Yes	Yes
FE	Yes	Yes	Yes
Observations	2,922,853	3,015,824	3,015,824
R^2^	0.741	0.737	0.737

Note: CV includes all unreported control variables as well as the constant term. FE represents fixed effects, including both firm-specific and province-year fixed effects. Unless explicitly stated, the following tables follow this note.

Given that Dell et al. (2014) [37] point out that the impact of climate on economic markets is multifaceted, controlling for economic variables may lead to “over-control” problems. Therefore, we alternatively control for the impact of city development trends by adding a city-year interaction term as a control variable in model (11). The result is shown in column (2) of Table 3, where we find that temperature still has an inverted U-shaped relationship with export product diversification. These results suggest that the omitted variable problem does not affect the basic conclusions of this study.

Subsequently, we address selection bias, encompassing both sample selection bias and self-selection bias. Our sample comprises all exporting firms registered in Chinese customs data, thereby minimizing sample selection bias. There are two problems with self-selection bias. Firstly, there is bias from unobserved firms that did not enter or exit the export market. Since data on the product categories of firms selling domestically are not available, we cannot investigate this issue empirically. However, logically, firms that do not enter or exit the export market are typically those that are eliminated due to lower competitiveness and risk resistance. Therefore, extreme temperatures are likely to have a greater negative impact on the diversification strategies of these firms, potentially underestimating the overall impact of temperature. Therefore, this bias doesn’t affect the conclusion of an inverted U-shaped relationship between temperature and export product diversification. Secondly, self-selection bias arises from firms’ location choices. Temperature-sensitive firms may prefer regions with comfortable temperatures when choosing a location. To address this, we examine the effect indirectly. Firms are likely to consider temperature conditions in different regions when selecting locations, suggesting a spillover effect of temperature across regions. To assess this, we use the inverse of city distances as weights to calculate the weighted average of temperature variables for other cities, constructing spatial lag terms for each temperature bin. The results in column (3) of Table 3 show that after controlling for this spillover effect, the study’s basic conclusion remains unchanged.

**Fig 2:**
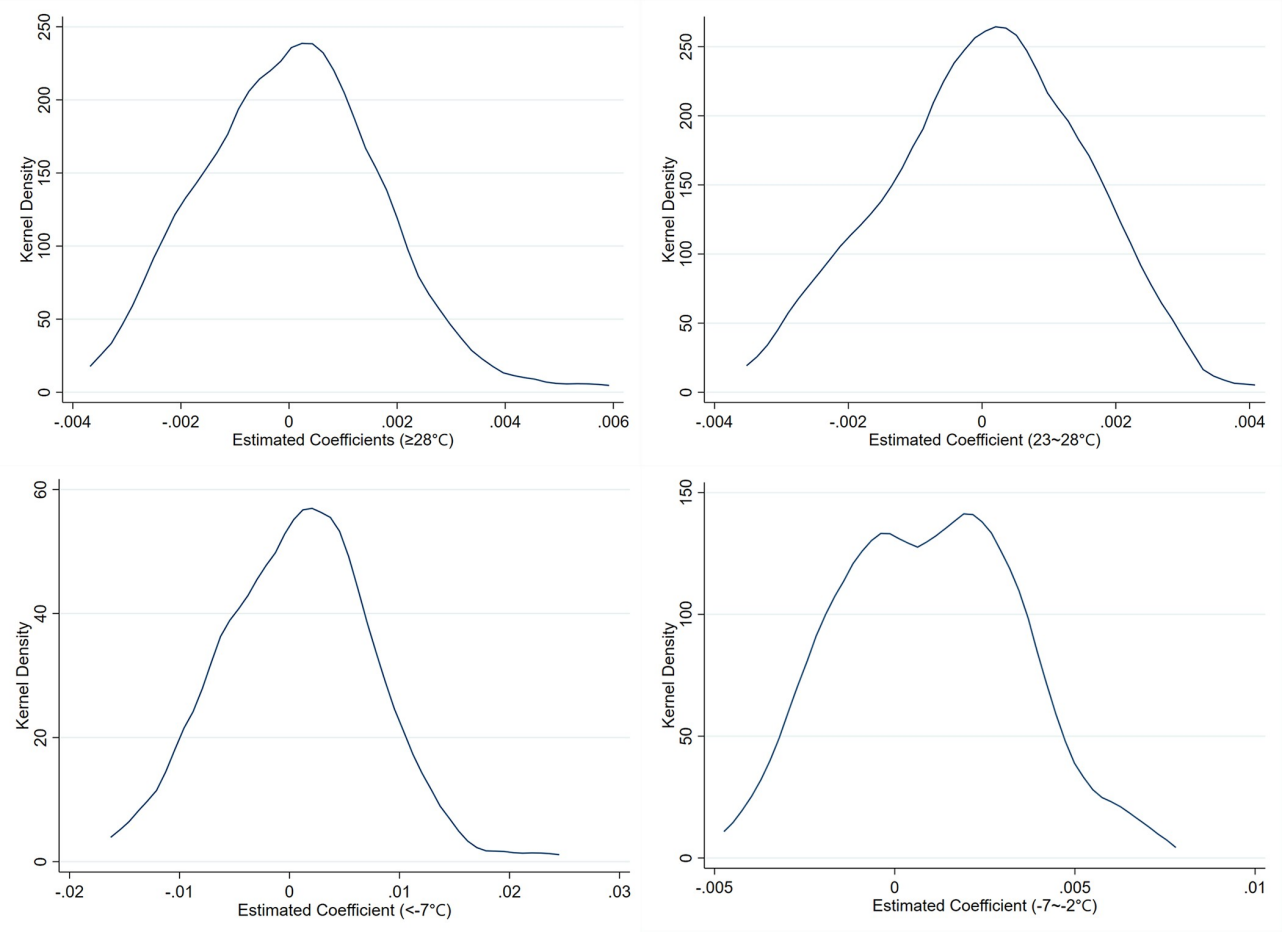
Placebo test. Fig 2 displays the research results of the four groups, which respectively illustrate the distribution of regression coefficients resulting from 100 random permutations for variables ≥28°C, 23~28°C, <-7°C, and -7~-2°C.

Finally, we turn our attention to the potential for bidirectional causality. In the time frame of our study (annual temperature distribution), temperature changes are primarily driven by natural factors and exhibit random fluctuations. In the existing literature, temperature is typically treated as an exogenous variable (Dell et al., 2014) [37]. However, caution is warranted regarding the possibility of unobservable factors that simultaneously affect both temperature and firm behavior, such as certain government environmental policies. To further address the impact of unobservable factors, we employ a placebo test by randomly shuffling the core explanatory variable 100 times and observing whether the temperature variable continues to significantly influence firms’ export decisions. A significant result would indicate the presence of unobservable factors, while an insignificant result would indicate the robustness of our conclusions. We run regressions after shuffling the variables for temperatures ≥28°C, 23~28°C, <-7°C, and -7~-2°C, respectively, and examine the distribution of the regression coefficients, as shown in Fig 2. The results show that after shuffling, the regression coefficients for both high and low temperatures are distributed around zero and are no longer significant, which is significantly different from the baseline regression results. This suggests that unobservable factors do not affect our conclusions, lending credibility to our findings.

### Model specification adjustment

In the baseline regression, we use an ordinary least squares (OLS) regression to examine the relationship between temperature bins and export product diversification. Given the count integer nature of the dependent variable (number of export product categories), we adopt a count model in this section and use negative binomial regression to re-evaluate the relationship between temperature bins and export diversification (see Table 4, column 1). The consistently negative regression coefficients across all temperature bins show an inverted U-shaped relationship, confirming the baseline findings. Notably, this baseline conclusion remains robust to a change in the econometric methodology. In addition, we explore the non-linear relationship between temperature and export product diversification using a non-parametric model in the benchmark regression setting. In this section, we construct the following commonly used parametric model to investigate the relationship between temperature and export product diversification.


$$Diversity_{icrt}=\alpha +\beta_{1}Temp_{icrt}\times Temp_{icrt}+\beta_{2}Temp_{icrt}+\delta_{1}X_{crt}+\gamma_{i}+\gamma_{rt}+\varepsilon_{icrt}$$ (12)

**Table 4:** Sensitivity analysis.

	(1)	(2)	(3)	(4)	(5)
Variables	Diversity	Diversity	Diversity	Diversity	Diversity
*Temp×Temp*		-0.0920**			
		(0.042)			
<-7°C	-0.0112***		-0.1082***	-0.1227*	-0.1339**
	(0.000)		(0.035)	(0.068)	(0.064)
-7~2°C	-0.0072***		-0.0885***	-0.0937*	-0.1213**
	(0.000)		(0.028)	(0.055)	(0.050)
-2~3°C	-0.0058***		-0.0644***	-0.0687*	-0.0859**
	(0.000)		(0.022)	(0.041)	(0.039)
3~8°C	-0.0029***		-0.0353*	-0.0361	-0.0492
	(0.000)		(0.018)	(0.034)	(0.034)
13~18°C	-0.0028***		-0.0136	-0.0076	-0.0078
	(0.000)		(0.017)	(0.013)	(0.023)
18~23°C	-0.0101***		-0.0872**	-0.0984**	-0.1024**
	(0.000)		(0.036)	(0.049)	(0.052)
23~28°C	-0.0094***		-0.0695**	-0.0855*	-0.0942**
	(0.000)		(0.030)	(0.044)	(0.046)
≥28°C	-0.0108***		-0.0664**	-0.0885**	-0.1347**
	(0.000)		(0.030)	(0.044)	(0.056)
Temp		2.1621			
		(1.327)			
CV	Yes	Yes	Yes	Yes	Yes
FE	Yes	Yes	Yes	Yes	Yes
Observations	3,015,824	3,015,824	3,015,824	3,015,824	3,015,824
R^2^		0.737	0.763	0.737	0.737

*Temp*_*irct*_ represents the annual average temperature of city c for firm *i* in year *t*, and the meanings of the other variables remain consistent with the previous text. The focus is on the regression coefficient *β*_1_. When *β*_1_ is significantly negative, it indicates an inverted U-shaped relationship between temperature and export product diversification, suggesting that both very low and very high temperatures inhibit firms’ diversification strategies. The regression results are presented in column (2) of Table 4, where *β*_1_ is indeed significantly negative, confirming the robustness of the baseline conclusion.

#### Outlier impact mitigation

To mitigate the potential influence of extreme values, we implement winsorization on the dependent variable, a technique that trims data points at the 0.5% and 99.5% percentiles. This process aims to reduce the influence of firms with extreme levels of diversification. The regression analysis using the modified sample data, based on Eq (11), is presented in Table 4, column (3). The results confirm the baseline conclusion, with statistically significant negative regression coefficients observed consistently across all temperature bins.

#### Changing the cluster level of standard errors

In the baseline regression, we account for the potential autocorrelation present in the error terms at both the firm and city-year levels. Subsequently, we refine our approach by relaxing this assumption and acknowledging the possibility of intra-city correlation among error terms. To address this, we adjust the standard errors to be clustered at the city level, as illustrated in Table 4, column (4). This slightly reduces the statistical significance, but maintains the inverted U-shaped relationship between temperature and export diversification. Most temperature bins pass statistical significance tests at the 10% level, underscoring the robustness of the baseline regression conclusions.

#### Weekday data examination

In accordance with Chinese labor laws, a two-day weekend system is implemented on Saturdays and Sundays, concentrating production and labor activities mainly on weekdays. Consequently, we focus on weekdays for a more nuanced analysis of temperature effects. Eq (11) is regressed again using temperature bin variables constructed from Monday to Friday data, as shown in Table 4 column (5). The results consistently show negative regression coefficients for all temperature bins, with the majority achieving statistical significance at the 5% level, consistent with previous analyses. Notably, the absolute values of the regression coefficients increase compared to the baseline, emphasizing that temperature predominantly affects export diversification mainly during weekdays.

#### Altering temperature bins length

**Fig 3:**
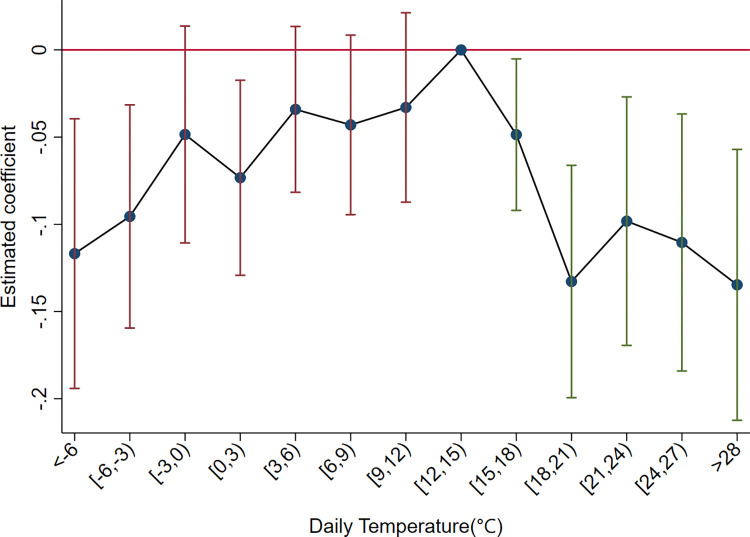
Impact of temperature on export diversification.

In the baseline regression, we divide temperature into 5°C bins. Here, we refine our assumption by assuming a uniform effect on export product diversification within 3°C temperature bins. Under this premise, we redefine temperature bins ranging from days below -6°C (*m* = 1) to days ≥27°C (*m* = 13). Specifically, if *m* = 1, it represents the number of days in a year with an average daily temperature below -6°C. *m* = 2 represents the days with an average daily temperature between -6 and -3°C, and so on. if *m* = 13, it means the days with daily average temperature ≥27°C. The regression with 12~15°C bin as the baseline group shows an inverted U-shaped relationship between temperature and export diversification. The regression coefficients in Fig 3 show a trend of initial increase and subsequent decrease with increasing temperatures, confirming the baseline conclusions. This confirms that the length of the temperature bin specified in the model does not affect the research results.

## Climate adaptation

Following the Industrial Revolution, rapid technological advances have provided multiple opportunities for climate adaptation. The widespread adoption of indoor work environments, coupled with innovations such as air conditioning and heating systems, enables individuals to regulate ambient temperatures, thereby increasing comfort and potentially productivity, mitigating the impact of climate variability. At the same time, improved financial systems, including insurance and credit, facilitate intertemporal risk management, thereby reducing the impact of climate-related risks. However, climate adaptation entails significant costs and necessitates a cognitive framework that aids in understanding and addressing the complexities of climate change. For Chinese exporters, the extent of adaptation to local temperature conditions and the factors influencing the ability to adapt to climate change remain key issues. In the following sections, we address these issues through the lens of firms’ export diversification.

### The impact of a firm’s export trade mode

China’s integration into the global value chain through its processing trade regime has been profound, significantly facilitating the transfer of industries from developed nations to developing nations and enhancing the optimal allocation of global resources. This integration has markedly propelled China’s export growth [38]. Within the realm of international trade research, the "productivity puzzle" of Chinese export firms has generated considerable debate. Studies have revealed that, excluding processing trade firms, the productivity of Chinese export firms surpasses that of non-export firms, thereby resolving the productivity puzzle. This suggests that Chinese processing trade firms possess unique characteristics; their engagement in exports is not predicated on higher productivity but rather on an order-based production model that revolves around "importing raw materials and exporting finished products." These firms are typically labor-intensive, exhibit low added value, and are highly substitutable, situating them at the lower end of the international value chain. In contrast, general trade firms tend to be capital-intensive, prioritize brand development, operate on a larger scale, exhibit higher productivity, and face less restrictive financing constraints (Manova and Yu, 2016) [39].

Acknowledging the pronounced differences between these firm types, our analysis categorizes the sample into two distinct groups: processing trade firms (*Ptrade* group) and general trade firms (*Gtrade* group), conducting separate regressions for each. The outcomes of the group regressions are detailed in columns (1) and (2) of Table 5-I. The analysis shows that processing trade firms are likely to reduce the variety of their export products when faced with extreme temperature events, whether hot or cold. This trend is less pronounced for general trade firms, which seem more inclined to maintain a diversified product strategy under cold temperature conditions. However, under high temperature conditions, general trade firms also suffer negative effects, but statistically, this is only evident at the 10% significance level in a negative correlation with export product diversity. In terms of economic significance, the effect on processing trade firms significantly exceeds that on general trade firms. The findings suggest that compared to general trade firms, processing trade firms have a weaker capacity to adapt to adverse climatic conditions, which may lead them to make more conservative strategic adjustments in terms of product diversification. In contrast, general trade firms appear to have developed more effective strategies to manage climate-related operational risks.

Considering the characteristics of processing trade firms mentioned earlier, we posit that the observed differences in temperature adaptability may stem from the following reasons. Processing trade firms often operate on lower profit margins, offer lower wages, and allocate relatively limited resources to research and development [40]. Such constrained profitability might restrict their ability to invest in climate adaptation measures, such as creating suitable working environments for workers or adjusting work schedules to accommodate temperature fluctuations. Moreover, their minimal investment in R&D could suggest a limited capacity for innovation in climate change adaptation technologies. This limitation can impede the adoption of new technologies and the formulation of responsive strategies, as well as restrict the firms’ flexibility to diversify their products and markets. As a result, in the face of increasingly frequent or severe extreme climatic conditions, processing trade firms may struggle to adapt their business strategies to sustain competitiveness. This challenging scenario could compel them to concentrate more on their existing, constrained product lines or markets to minimize the need for new investments and R&D. These findings highlight that processing trade firms represent a critical vulnerability in the climate adaptation of Chinese export firms. Future strategies should aim to assist these firms in enhancing worker conditions, upgrading production equipment with advanced technologies such as digitization, and instituting monitoring systems to decrease machinery sensitivity to temperature. Such measures will foster the evolution of green and sustainable production practices.

### Impact of regional financial development

Access to finance is pivotal for firms seeking to bolster their resilience against extreme climate events [41,42]. In regions with immature financial systems, financing channels are often limited, the cost of borrowing for firms is higher, and the amount of financing available is lower. These constraints make it harder for firms to get the support they need to cope with sudden economic pressures, such as those caused by extreme climate events [43]. Furthermore, financial constraints may prevent timely adjustments to production processes or supply chains, limiting their ability to maintain or expand a diverse range of products and services in the face of extreme temperature changes. In addition, these financial constraints may limit the ability of firms to develop new products or services to respond to market shifts, thereby reducing their adaptability and survival in extreme climatic conditions. However, the extent and speed with which firms can access finance is influenced by a number of factors, of which the external financial environment is a key one. The local financial environment has a direct impact on the efficiency and economy with which firms can secure external finance. Regions with more advanced financial systems often have a larger number of financial institutions and more robust capital liquidity, offering firms more efficient and cost-effective financing options. As a result, we expect the negative impact of extreme temperatures on firms’ diversification strategies to be less pronounced in cities with mature financial systems.

To empirically examine this hypothesis, we employ the ratio of city commercial bank branches to total financial institution branches as an indicator of a city’s financial development level. City commercial banks reflect the maturity of urban financial systems in three ways: First, they possess greater autonomy than larger banks in approving loans to small and micro firms, which aids in expanding credit supply and equalizing the distribution of financial resources, thus reducing financing costs; Second, as local financial institutions, they have a deeper understanding of local economic conditions and market situations, effectively reducing information asymmetry and providing higher quality credit services; Third, the competition they introduce stimulates the financial industry, enhancing the availability of financing channels for firms and alleviating capital constraints. Based on the median level of financial development, cities are divided into two categories: those that are less developed (*Underdevelope* group) and those with advanced financial systems (*Advance* group). A regression analysis is then carried out (as shown in columns (3) and (4) of Table 5-I). The results show that in cities with less developed financial systems, extreme temperatures significantly inhibit the firms’ diversification strategies in export markets. In contrast, this negative effect is significantly reduced in cities with a mature financial infrastructure. This finding highlights the importance of local financial development in enhancing firms’ strategic flexibility and risk management in the context of climate change, particularly in terms of resource allocation and mitigation of economic shocks.

**Table 5:** I. Climate adaptation. II. Climate adaptation. Ш. Climate adaptation.

	(1)	(2)	(3)	(4)
Variables	Ptrade	Gtrade	Underdevelope	Advance
<-7°C	-0.2423***	-0.0083	-0.2419***	-0.0019
	(0.076)	(0.026)	(0.075)	(0.032)
-7~2°C	-0.1422***	-0.0146	-0.1516**	-0.0184
	(0.055)	(0.018)	(0.061)	(0.023)
-2~3°C	-0.1062**	-0.0038	-0.1090**	-0.0109
	(0.042)	(0.015)	(0.043)	(0.017)
3~8°C	-0.0526*	-0.0056	-0.0725**	0.0096
	(0.030)	(0.011)	(0.034)	(0.012)
13~18°C	0.0072	0.0183	0.0003	-0.0277**
	(0.025)	(0.019)	(0.030)	(0.013)
18~23°C	-0.1533**	-0.0271	-0.1593***	-0.0055
	(0.063)	(0.019)	(0.058)	(0.018)
23~28°C	-0.1271**	-0.0515*	-0.1252***	-0.0101
	(0.052)	(0.027)	(0.046)	(0.021)
≥28°C	-0.1258**	-0.0630*	-0.1325***	-0.0443*
	(0.054)	(0.034)	(0.047)	(0.024)
CV	Yes	Yes	Yes	Yes
FE	Yes	Yes	Yes	Yes
Observations	1,630,475	1,105,352	1,502,227	1,464,566
R^2^	0.799	0.754	0.733	0.772
	**(1)**	**(2)**	**(3)**	**(4)**
**Variables**	**Developing**	**Developed**	**Lenergy**	**Henergy**
<-7°C	-0.0290	-0.9541***	-61.4848**	-0.1229
	(0.038)	(0.262)	(25.898)	(0.184)
-7~2°C	-0.0205	-0.6688***	-0.6884	-0.1681
	(0.024)	(0.199)	(0.459)	(0.154)
-2~3°C	-0.0288*	-0.4899***	-0.5962**	-0.1130
	(0.016)	(0.154)	(0.237)	(0.144)
3~8°C	0.0085	-0.4106***	-0.5227***	0.0529
	(0.013)	(0.134)	(0.172)	(0.112)
13~18°C	0.0162	-0.0185	0.0265	-0.2330**
	(0.012)	(0.079)	(0.092)	(0.113)
18~23°C	0.0091	-0.2476**	-0.2500*	-0.2280
	(0.016)	(0.121)	(0.142)	(0.148)
23~28°C	0.0122	-0.2516**	-0.2647	-0.2070
	(0.018)	(0.122)	(0.165)	(0.134)
≥28°C	0.0039	-0.4037***	-0.4060**	-0.1417
	(0.019)	(0.144)	(0.165)	(0.124)
CV	Yes	Yes	Yes	Yes
Firm FE	Yes	Yes	Yes	Yes
Observations	1,505,678	1,463,285	882,233	561,632
R^2^	0.703	0.760	0.776	0.748
	**(1)**	**(2)**	**(3)**	**(4)**
**Variables**	**Northern Cities**	**Southern Cities**	**Cold Cities**	**Hot Cities**
<-7°C	-0.0281	0.0711	-0.0372	3.2633
	(0.042)	(0.161)	(0.037)	(2.762)
-7~2°C	-0.0143	-0.0636	-0.0213	0.0346
	(0.031)	(0.064)	(0.026)	(0.263)
-2~3°C	0.0070	-0.0971***	-0.0174	-0.0882
	(0.025)	(0.037)	(0.020)	(0.055)
3~8°C	0.0236	-0.0564**	0.0007	-0.0497
	(0.022)	(0.028)	(0.016)	(0.040)
13~18°C	-0.0000	-0.0142	0.0149	-0.0292
	(0.024)	(0.024)	(0.017)	(0.032)
18~23°C	0.0225	-0.1361***	0.0298	-0.1737***
	(0.030)	(0.050)	(0.023)	(0.061)
23~28°C	0.0145	-0.1174***	0.0079	-0.1464***
	(0.032)	(0.042)	(0.025)	(0.050)
≥28°C	0.0016	-0.1198***	-0.0055	-0.1475***
	(0.032)	(0.042)	(0.027)	(0.049)
CV	Yes	Yes	Yes	Yes
Firm FE	Yes	Yes	Yes	Yes
Observations	823,240	2,192,584	1,615,926	1,395,795
R^2^	0.671	0.750	0.714	0.751

### Impact of economic development level and energy usage

The previous analysis has shown that a higher level of financial development in cities can enhance firms’ adaptability to extreme temperatures by providing greater access to capital and more sophisticated financial services. However, a potential concern is that financial development may be closely related to the city’s economic development, potentially masking the effects attributed to the financial system alone. To investigate this, the sample is segmented into less developed cities (*Developing* group) and developed cities (*Developed* group) based on the median gross domestic product (GDP) of the firms’ locations, and regressions are run for each group. The results are shown in columns (1) and (2) of Table 5-II. The regression results indicate that firms in developed cities are more likely to reduce the diversity of export products under extreme temperatures, while those in less developed cities tend to maintain or even increase their diversification strategies, with some temperature conditions promoting a positive effect. This suggests that the positive moderating role of financial development is not driven by the level of economic development. Conversely, economic growth does not seem to improve the adaptability of local firms to extreme disasters.

To clarify why firms in more developed cities are more affected, the sample of these cities is further divided into low-energy consumption cities (*Lenergy* group) and high-energy consumption cities (*Henergy* group), using the median of energy consumption per unit of GDP as a criterion. This indicator is derived from provincial energy consumption divided by provincial GDP, reflecting energy consumption per unit of economic output. Since adaptation to extreme temperatures for businesses and residents often relies on energy-intensive tools such as air conditioning or heating systems, it was hypothesized that higher energy consumption could facilitate adaptation to extreme temperatures, potentially offsetting the negative regulatory effects of temperature impacts on economically advanced cities. Specifically, regressions were run separately for high and low-energy consumption cities within the developed city category. The results, presented in columns (3) and (4) of Table 5-II, show that in areas with lower energy consumption, the diversification of firms’ export products decreases significantly, while the opposite is true for areas with higher energy consumption, suggesting that energy consumption enhances the ability of firms to adapt to extreme temperatures.

### Impact of firms’ location

Scholars generally believe that short-term impacts can be mitigated in the long term through behavioral adjustments of economic agents, leading to adaptability (Hornbeck, 2012). This study delves into the development of temperature adaptability across different regions in China, a nation characterized by substantial latitude and temperature gradients. Specifically, we scrutinize whether northern China, which experiences lower average temperatures and frequent cold weather, has greater adaptability to low-temperature shocks, and whether southern China, with its higher average temperatures and frequent hot weather, is more adept at coping with high-temperature shocks.

Our analysis classifies Chinese cities into southern and northern regions based on economic geography. The southern regions include Shanghai, Jiangsu, Zhejiang, Anhui, Fujian, Jiangxi, Hubei, Hunan, Guangdong, Guangxi Zhuang Autonomous Region, Hainan, Chongqing, Sichuan, Guizhou, Yunnan, and Tibet Autonomous Region. The northern regions include Beijing, Tianjin, Hebei, Shanxi, Inner Mongolia Autonomous Region, Liaoning, Jilin, Heilongjiang, Shandong, Henan, Shaanxi, Gansu, Qinghai, Ningxia Hui Autonomous Region, and Xinjiang Uygur Autonomous Region. This analysis excludes Hong Kong, Macau, and Taiwan. We then conduct grouped regressions, as shown in Table 5-Ш. Column (1) shows results for firms in northern cities, where both low and high temperatures exert insignificant impacts. Column (2) presents results for firms in southern cities, which are the epicenter of China’s trade firms and the primary driver of our findings. High temperatures are found to significantly diminish the diversity of export products, suggesting that southern cities have not yet achieved effective adaptation to high temperatures. Considering China’s current economic geography, marked by a more robust southern region and a relatively weaker northern counterpart, this conclusion underscores that even firms in developed regions are poorly adapted to climate shifts. Southern cities, being vital to China’s foreign trade, ought to prioritize strategies for preparing for escalating temperatures and increasing extreme heat events. This can be accomplished by investing in climate-adaptive infrastructure and guiding firms to enhance their production environments, thereby promoting sustainable development. Our findings are in harmony with those of Zhang and Li (2023) and Burke et al. (2015), who observed that regions with warmer climates or higher economic wealth have not implemented substantial adaptive measures, with economic indicators remaining sensitive to high-temperature shocks.

Furthermore, aligning with the methodology of Zhang and Li (2023), we categorize cities as historically hot or cold based on their average annual temperature from 1970 to 1999. Cities that surpass the national median temperature are designated as hot, while those below it are labeled as cold, with regressions conducted for each group. Columns (3) and (4) of Table 5-Ш reveal that in historically hot cities, an upsurge in the frequency of high-temperature days is significantly associated with a reduction in export product diversity. Conversely, in historically cold cities, an increase in the frequency of low-temperature days tends to reduce export product diversity, although this effect does not achieve statistical significance. These results are consistent with those of columns (1) and (2), indicating that Chinese export firms have not yet implemented significant adaptive adjustments to temperature fluctuations. As climate change intensifies and high temperatures, particularly extreme heat, become more prevalent, southern Chinese cities are poised to confront more severe repercussions. It is imperative that governments and firms in these regions prioritize strategic adjustments to augment their climate adaptability.

## Structural factors in export diversification decline

In this section, we analyze the factors that influence the reduction in the variety of product categories exported by firms due to extreme temperatures. We adopt a dynamic perspective, focusing on three key aspects: product entry, survival, and exit.

Specifically, the number of product categories exported by firms can be decomposed into two main components:

$$Diversity_{it}=\sum_{p}New_{ipt}+\sum_{p}Exist_{ipt}$$ (13)

Where *i*, *p*, and *t* denote the firm, product, and year respectively. *Diversity* reflects the export product categories of the firms. *New*_*ipt*_ is equal to 1 if product *p* belongs to a new category. Otherwise, it is equal to 0. Here, new category denotes products that were absent in the previous year but are present in the current year. *Exist*_*ipt*_ is equal to 1 if the product *p* belongs to an existing category. Otherwise it is equal to 0. Where, exist category refers to products that exist in the previous year and continue to exist in the current year. Eq (13) expresses that the total number of product categories exported by a firm in a given year is equal to the sum of the new categories added and the number of categories surviving from the previous year. Therefore, on the basis of Eq (13), we will examine how temperature affects the number of new product categories and the number of existing product categories in the export of firms.

**Fig 4:**
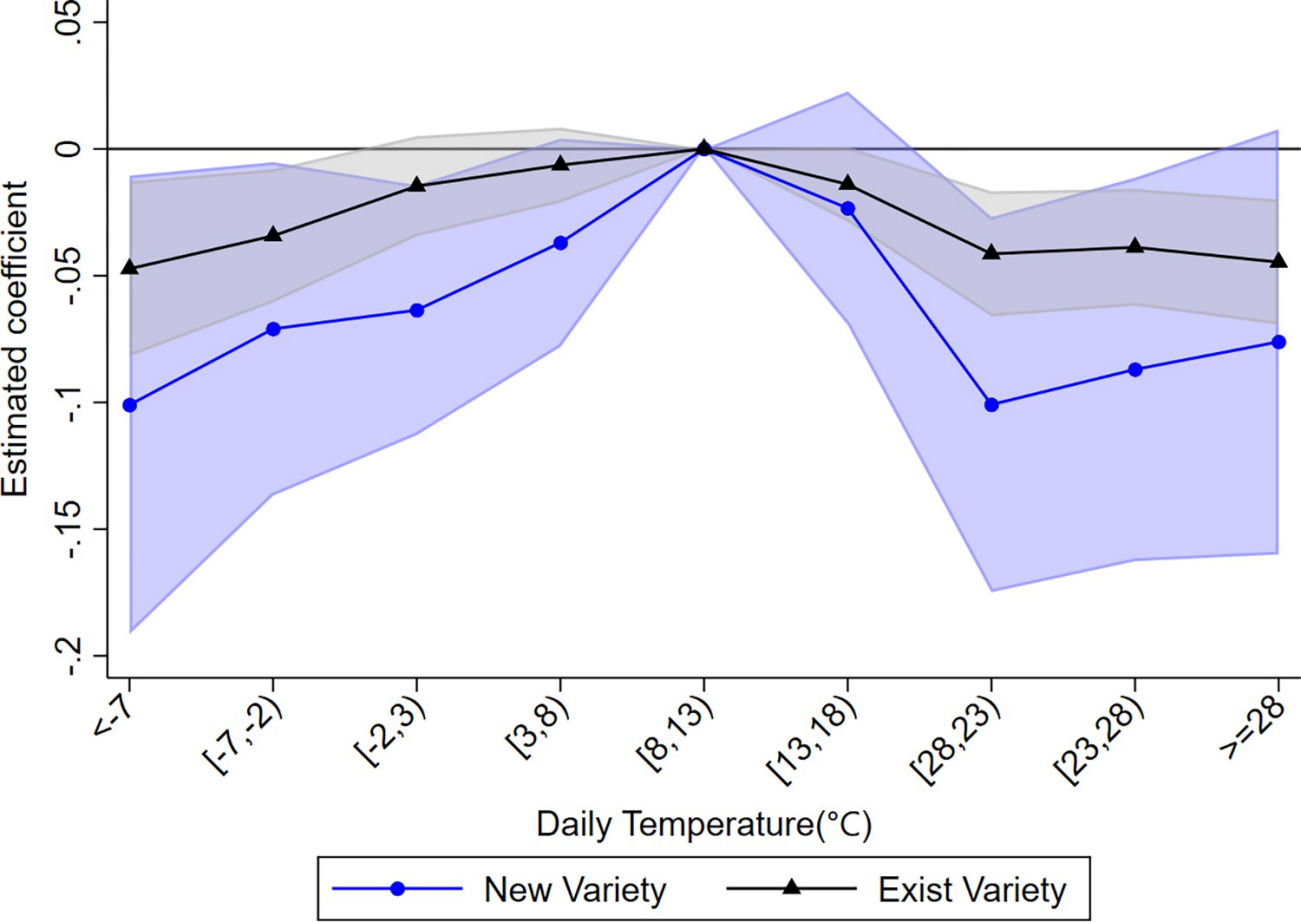
Temperature and dynamics of firm exported products. Solid circles indicate the effect of temperature on newly added product categories, while triangles indicate its effect on existing product categories. The shaded area includes the 90% confidence interval.

We replaced the dependent variables in Eq (11) with the number of newly added and existing product categories in firm exports, respectively, for the regression analysis, yielding the results shown in Fig 4. Solid circles indicate the effect of temperature on newly added product categories, while triangles indicate its effect on existing product categories. The shaded area includes the 90% confidence interval, with blue and grey shading indicating the confidence intervals for newly added and existing product categories respectively. The regression results show an inverted U-shaped relationship between temperature and both newly added and existing product categories. The results suggest that extreme temperatures reduce the product diversification of firms’ exports by reducing the variety of both newly added and existing products. Looking at the marginal effects, temperature bins have a more pronounced negative effect on newly added product categories than on existing ones. The addition of new products, which indicates positive development and expansion, is hindered by unfavorable temperatures, which poses a challenge to the long-term sustainable growth of firms.

In addition, a dynamic decomposition of changes in the number of product categories exported by firms can be expressed in the following formula:

$$\Delta Diversity_{i,t}=\sum_{p}N{\mathrm{ew}}_{ip,t}-\sum_{p}Exit_{ipt}$$ (14)

Where *Exit*_*ipt*_ equals 1 if the product *p* belongs to an exit category, otherwise it is 0. Exit category refers to products that were present in the previous year but are absent in the current year. The remaining variables are consistent with the above context. Eq (14) shows that the change in the number of product categories this year is equal to the number of new product categories minus the number of exiting product categories. This dynamic change in product exits, as indicated by Eq (14), influences the second-order changes in the number of product categories exported by firms. In this section, we examine the impact of temperature on product exits in firms’ exports using two different approaches.

We use Eq (11) to regress the influence of temperature on the number of product categories that firms exit from the export market, represented by the blue dots in Fig 4. These dots represent the impact of the temperature bins on the exiting product categories. In particular, temperatures above the optimal range tend to increase the number of product categories exiting the export market, while the opposite trend is observed when temperatures fall below the optimal range. However, the effects in the lowest and highest temperature bins lack statistical significance. Consequently, there is no conclusive evidence indicating that extreme temperatures reduce the number of exported product categories by encouraging product exits.

In addition, we examine this issue in more detail using data at the firm-product level. We build the following econometric model:

$$Exit_{ipcrt}=\alpha +\sum_{m\neq 5}\beta_{1}^{m}Tbin_{crt}^{m}+\delta_{1}X_{crt}+\gamma_{i}+\gamma_{pt}+\gamma_{rt}+\varepsilon_{ipcrt}$$ (15)

**Fig 5:**
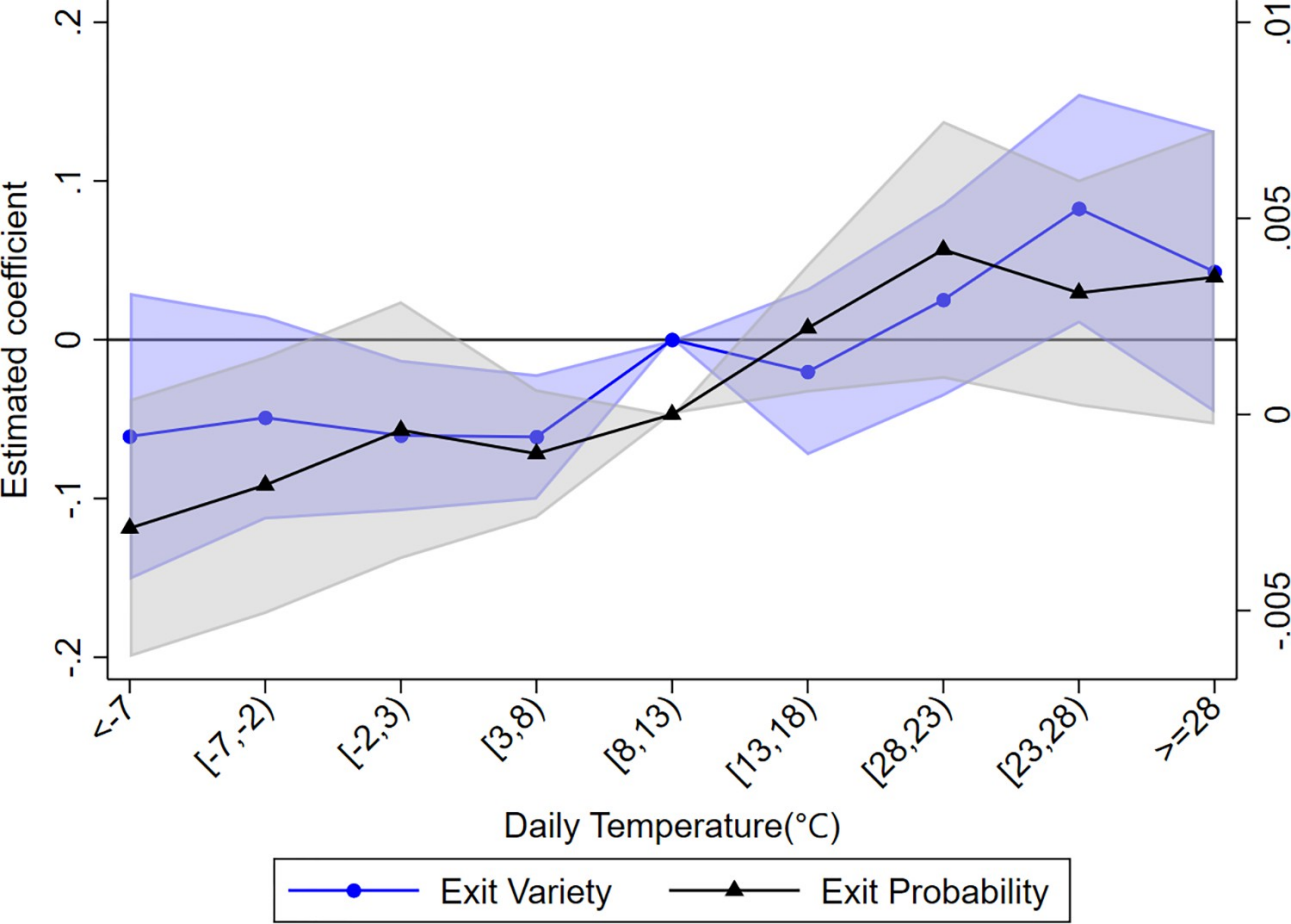
Temperature and product exits. The dots depict the impact of the temperature bins on the exiting product categories, corresponding to the values on the left-hand axis. The triangles. depict the impact of the temperature bins on the probability of products exiting the export market, corresponding to the values on the right-hand axis.

Where, *Exit*_*ipcrt*_ indicates whether product *p* of firm *i* has exited the export market in year *t*. If the product has exited, the value of *Exit*_*ipcrt*_ is 1; otherwise it is 0. The other variables retain their meaning from the previous context. The regression results, shown by the black triangles in Fig 5 (corresponding to the right axis), indicate the influence of temperature bins on the probability of products exiting the export market. The results are consistent with the effect on the number of exiting product categories. Specifically, temperatures above the optimal range tend to increase the probability of products exiting the export market, while the opposite is observed for temperatures below the optimal range. However, statistically, the effects in the low-temperature bins lack significance below a 10% threshold, while the effects in the high-temperature bins show some significance. Overall, temperatures above the optimal range moderately increase the likelihood of products leaving the export market.

## Extensive research

In the benchmark regression, our focus is on assessing the influence of temperature on firms’ export product diversification. Another important aspect of diversification in export strategies is the diversification of export destinations [44]. Given the increasingly complex global political and economic landscape, strategic decisions regarding the international distribution of sales networks have become paramount. Like product diversification, export destinations diversification serves as a crucial risk mitigation strategy for firms [45]. However, due to the associated entry costs, firms face limitations in expanding their export destinations.

Typically, firms with higher productivity are better able to cover market entry costs, allowing them to develop additional export markets. Conversely, as productivity declines, firms may choose to minimize costs by abandoning certain export markets. Building on previous studies [27], we expect that deviations from the optimal temperature, which tend to reduce firm productivity, will lead firms to reduce their export destinations, resulting in a lower degree of diversification. We then empirically test this hypothesis using the following econometric model.


$$Country_{icrt}=\alpha +\sum_{m\neq 6}\beta_{1}^{m}Tbin_{crt}^{m}+\delta_{1}X_{crt}+\gamma_{i}+\gamma_{rt}+\varepsilon_{icrt}$$ (16)

**Fig 6:**
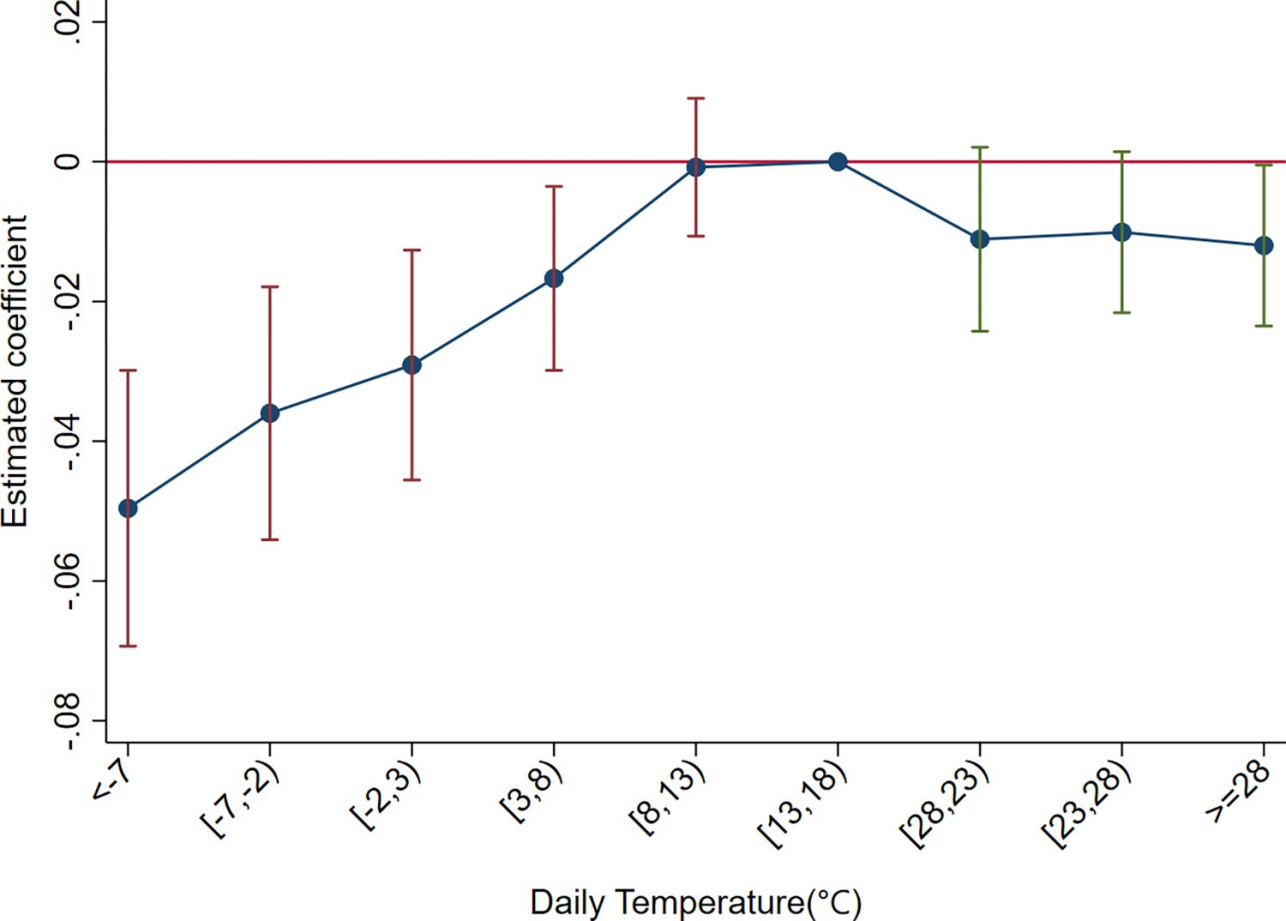
The effects of temperature on firms’ export destination.

The variable *Country*_*icrt*_ denotes the number of destinations of the firms’ exports, while the other variables retain their meanings consistent with the previous context. We refer to the temperature bin 13~18°C as the baseline group for the regression, as the empirical analysis identified it as the optimal category. The regression results are presented in Fig 6, where the solid dots represent the regression coefficients for each temperature bin, accompanied by their respective 90% confidence intervals. The results show that the regression coefficients for all temperature bins are negative, implying that destination diversification has an inverted U-shaped relationship with the temperature bins, suggesting that as temperatures deviate further from the optimal range, there is a greater reduction in the number of destinations for firms’ exports. In particular, the impact of extremely low temperatures is more pronounced and economically significant than that of high temperatures.

**Fig 7:**
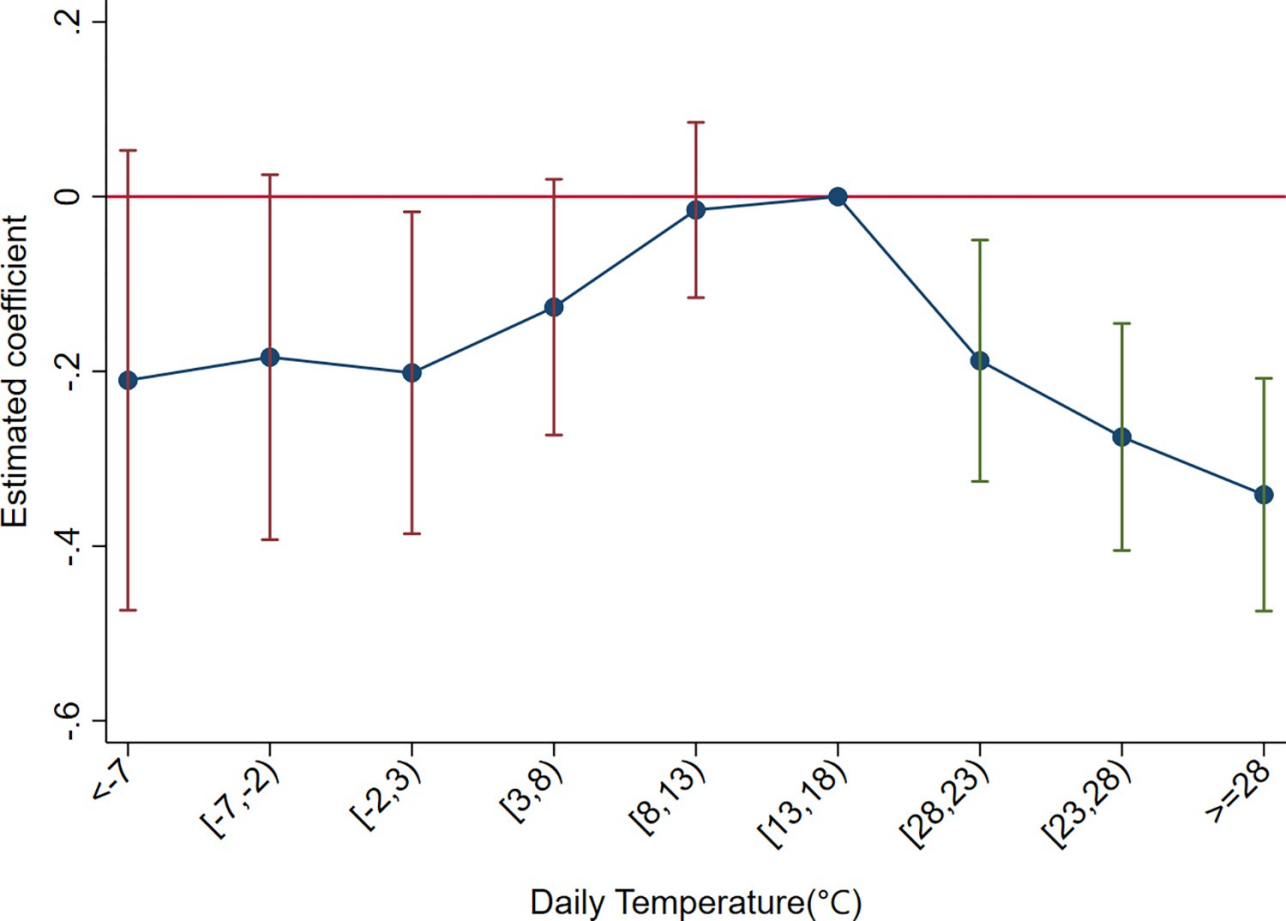
The effects of temperature on firms’ export relationship diversification.

Further analysis examines the influence of temperature on the diversification of firms’ export relationships. The diversification of relationships is defined as the number of country-product pairs for exports. When this variable is substituted into Eq (16) for the regression, the results presented in Fig 7 show an inverted U-shaped pattern. In contrast to the effect on export destinations, the high-temperature bins yield more significant coefficients, indicating a significant reduction in firms’ export relations as the positive deviation from the optimal temperature increases. In the low-temperature bins, only the coefficient for -2~3°C passes a 10% significance test. The above results suggest an inverted U-shaped relationship between temperature and the diversification of firms’ export destinations and relationships, but characterized by an asymmetric effect between high and low temperatures.

## Discussion

Previous studies have revealed a nonlinear, inverted U-shaped relationship between temperature and economic output at both macro and micro levels. Specifically, micro-level research indicates that extreme temperatures reduce industrial firms’ output, productivity, export volumes, and product quality. Using non-parametric models, this paper examines how temperature influences export firms’ diversification strategies. Unlike prior research that focused on the scale of firms or economies, this study delves into how extreme temperatures affect the variety of exported products, export partners, and relationships. This provides a deeper understanding of the multidimensional impacts of extreme temperatures on economic behavior and their profound economic implications. The findings reveal that export firms adjust their diversification strategies in response to frequent extreme temperatures, affirming the inverted U-shaped relationship between temperature and export diversification. Our study complements previous micro-level climate economics research by emphasizing export diversification. These findings have significant practical implications for enhancing firms’ export market diversity and competitiveness and promoting climate adaptability among firms and society.

Our research uncovers that temperature shocks, both high and low, are associated with a diminution in the variety of export products among firms. Specifically, for a prototypical firm, an increment of one day with temperatures below -2°C is correlated with a decrement of 0.22 in the count of export product categories, aggregating to a yearly reduction of 2.94 categories. Similarly, an increment of one day with temperatures at or above 28°C results in a 0.09 decrease, culminating in an annual reduction of 4 categories. These results demonstrate robustness after accounting for endogeneity concerns, utilizing alternative methodological approaches, addressing outlier effects, refining the hierarchy of standard error clustering, and reconfiguring temperature bins through diverse methodologies. By dynamically decomposing products, our findings indicate that extreme temperature shocks reduce the number of export product varieties primarily by decreasing the number of new and continuing product varieties. However, the exit of products does not emerge as a predominant catalyst. Positive deviations from the optimal temperature marginally enhance product exits, thus diminishing export product diversity. In contrast, negative deviations do not incite product exits and exert a dampening influence under certain temperature conditions. Furthermore, we also find that temperature exhibits an inverted U-shaped relationship with both the number of export destination countries and the number of export relationships.

Upon delving deeper into the types of firms that exhibit climate adaptability, our research indicates that general trade firms are more resilient to extreme temperatures compared to processing trade firms. This enhanced resilience may stem from their robust profitability, elevated wage structures, and substantial investment in research and development (R&D), which collectively enable them to furnish superior working conditions and allocate resources towards climate adaptation initiatives. Firms located in regions with developed financial markets demonstrate greater adaptability to extreme temperatures. However, a higher level of economic development in a city does not necessarily enhance firms’ adaptability unless accompanied by high energy use. Finally, our findings suggest that firms have not yet fully adapted to the local climate conditions, implying that climate adaptation strategies are not integrated into firms’ decision-making frameworks. Furthermore, there may be an absence of adequate climate adaptation infrastructure within certain cities. This disparity is particularly pronounced in southern Chinese cities, which play a pivotal role in export trade and are frequently subjected to high temperatures. Despite this, the firms in these regions have not demonstrated a sufficient level of adaptability to thermal extremes.

The study has some limitations that need to be further explored in the future. Firstly, our empirical study is constrained by the scope of available data, which encompasses the period from 2000 to 2016 and represents the most recent and extensive dataset of firm-level export information in China. However, given the rapid development of digital technology and artificial intelligence, it is worth exploring whether the conclusions of this study remain valid or have been subject to considerable alteration. Future research would be enriched by accessing more current and granular data, allowing for a deeper investigation into these phenomena. Research efforts could concentrate on evaluating recent data to discern whether the influence of prevailing extreme temperatures has been exacerbated or ameliorated through the digital technology revolution or advancements in artificial intelligence. Undertaking such an updated analysis would not only deepen our comprehension of the impact of climate change but also guide the development of strategies that harness contemporary technology to adapt to these shifts. Secondly, our study shows that extreme temperatures affect firms’ diversification strategies and that certain factors could potentially augment firms’ adaptability to temperature changes, thereby reducing the impact. Future research could further systematically investigate which factors play a more crucial role in enhancing the climate adaptability of firms and how they operate.

## Conclusion

Building on a theoretical framework, this study investigates the relationship between temperature and firm diversification in the export market using China’s daily meteorological data and detailed customs export data from 2000 to 2016. Employing a non-parametric model, we identify an inverted U-shaped relationship between temperature and export product diversification. In the analysis of climate adaptability, general trade firms demonstrate greater adaptability to extreme temperatures compared to processing trade firms. Firms located in cities with developed financial markets also tend to exhibit stronger adaptability to extreme temperatures. However, a high degree of urban economic development does not necessarily enhance local firms’ adaptability to extreme temperatures. Energy consumption, on the other hand, can enhance the adaptive capacity of firms in developed regions. Lastly, the study observes that firms have not yet fully adapted to local climatic norms, especially in southern regions, which serve as important trade hubs and remain highly sensitive to heat shocks. A dynamic decomposition of firms’ export products reveals that extreme temperatures reduce the variety of export products primarily by decreasing the number of newly introduced and existing product categories. Moreover, the research discerns an inverted U-shaped relationship between temperature and the diversification of both firms’ export destinations and relationships.

The findings of this study yield significant policy implications for both corporate entities and governmental bodies. Initially, there is an imperative for the establishment of capacity-building initiatives focused on fortifying the resilience of firms, with a particular emphasis on processing trade firms. This could encompass educational programs and training modules on the assessment and management of climate-related risks. At the onset of 2024, China’s Ministry of Commerce, in conjunction with other governmental departments, introduced a suite of 12 measures to enhance the development of processing trade, aiming to create a "Processing Trade 2.0" and accelerate the transition of firms from OEM (Original Equipment Manufacturing) to ODM (Original Design Manufacturing) and OBM (Original Brand Manufacturing). Processing trade firms should leverage these policy initiatives to gradually upgrade and transform, providing more competitive work environments and salaries for labor, focusing on green and sustainable development, and proactively investing in climate adaptability. This will enable them to achieve greater autonomy and strategic flexibility in international competition.

Secondly, the government needs to accelerate the construction of climate-adaptive cities, particularly implementing high-temperature adaptation measures in southern cities. This initiative should encourage the southern regions to invest more in infrastructure designed to withstand extreme heat and gradually increase fiscal expenditure on high-temperature adaptation. It should also use financial mechanisms to incentivize firms to contribute to climate resilience. For example, Encouraging green finance initiatives can help direct capital towards sustainable practices and technologies. This could involve creating financial instruments that favor environmentally friendly investments. Policies should support R&D in climate-resilient technologies and practices. This could include public-private partnerships to innovate in areas such as energy efficiency, sustainable materials, and smart logistics.

Lastly, cognizant of the inherently global dimensions of trade, international cooperation is essential to address climate change. Policies should support global initiatives aimed at reducing emissions and promoting climate adaptation. By considering these policy implications, governments can play a proactive role in helping their economies transition to a more climate-resilient future, ensuring that the economic impacts of climate change are mitigated and that businesses, especially export firms, can continue to thrive in an increasingly unpredictable climate landscape.

## References

[1] Bohra-Mishra P, Oppenheimer M, Hsiang S M. Nonlinear permanent migration response to climatic variations but minimal response to disasters. Proceedings of the National Academy of Sciences. 2014; 111(27): 9780–9785. doi: 10.1073/pnas.1317166111 24958887 PMC4103331

[2] Huang K, Zhao H, Huang J, et al. The impact of climate change on the labor allocation: Empirical evidence from China. Journal of Environmental Economics and Management. 2020; 104: 102376.

[3] Ouyang Y, Sun C, Wei X, et al. Will temperature changes in the host country reduce the inflow of international direct investment? Micro evidence from Chinese listed companies. Environmental and Resource Economics. 2023; 1–26.

[4] Deschênes O, Greenstone M, Guryan J. Climate change and birth weight. American Economic Review. 2009; 99(2): 211–17. doi: 10.1257/aer.99.2.211 29505213

[5] Fishman R, Carrillo P, Russ J. Long-term impacts of exposure to high temperatures on human capital and economic productivity. Journal of Environmental Economics and Management. 2019; 93: 221–238.

[6] Braguinsky S, Ohyama A, Okazaki T, et al. Product innovation, product diversification, and firm growth: Evidence from Japan’s early industrialization. American Economic Review. 2021; 111(12): 3795–3826.

[7] Lendle A, Vézina P L. Internet technology and the extensive margin of trade: evidence from ebay in emerging economies. Review of Development Economics. 2015; 19(2): 375–386.

[8] Mayer T, Melitz M J, Ottaviano G I P. Market size, competition, and the product mix of exporters. American Economic Review. 2014; 104(2): 495–536.

[9] Lu Y, Tao Z, Zhang Y. How do exporters adjust export product scope and product mix to react to antidumping? China Economic Review. 2018; 51: 20–41.

[10] Melitz M J. The impact of trade on intra‐industry reallocations and aggregate industry productivity. Econometrica. 2003; 71(6): 1695–1725.

[11] Bernard A B, Redding S J, Schott P K. Multiple-product firms and product switching. American Economic Review. 2010; 100(1): 70–97.

[12] Nocke V, Yeaple S. Globalization and multiproduct firms. International Economic Review. 2014; 55(4): 993–1018.

[13] Nocke V, Schutz N. Multiproduct‐firm oligopoly: An aggregative games approach, Econometrica. 2018; 86(2): 523–557.

[14] Mayer T, Melitz M J. Ottaviano G I P. Product mix and firm productivity responses to trade competition. Review of Economics and Statistics. 2021; 103(5): 874–891.

[15] Dimitrov V, Tice S. Corporate diversification and credit constraints: Real effects across the business cycle. The Review of Financial Studies. 2006; 19(4): 1465–1498.

[16] Chatterjee A, Dix-Carneiro R, Vichyanond J. Multi-product firms and exchange rate fluctuations. American Economic Journal: Economic Policy. 2013; 5(2):77–110.

[17] Manova K, Wei S J, Zhang Z. Firm exports and multinational activity under credit constraints. Review of Economics and Statistics. 2015; 97(3): 574–588.

[18] Abeberese A B, Chen M. Intranational trade costs, product scope and productivity: Evidence from India’s Golden Quadrilateral project. Journal of Development Economics. 2022; 156: 102791.

[19] Jones B F, Olken B A. Climate shocks and exports. American Economic Review. 2010; 100(2): 454–59.

[20] Dell M, Jones B F, Olken B A. Temperature shocks and economic growth: Evidence from the last half century. American Economic Journal: Macroeconomics. 2012; 4(3): 66–95.

[21] Burke M, Hsiang S M, Miguel E. Global non-linear effect of temperature on economic production. Nature. 2015; 527(7577): 235–239. doi: 10.1038/nature15725 26503051

[22] Dallmann I. Weather variations and international trade. Environmental and Resource Economics. 2019; 72(1): 155–206.

[23] Linsenmeier M. Temperature variability and long-run economic development. Journal of Environmental Economics and Management. 2023, 121: 102840.

[24] Chen X, Yang L. Temperature and industrial output: firm-level evidence from China. Journal of Environmental Economics and Management. 2019; 95: 257–274.

[25] Addoum J M, Ng D T, Ortiz-Bobea A. Temperature shocks and establishment sales. The Review of Financial Studies. 2020; 33: 1331–1366.

[26] Pankratz N, Bauer R, Derwall J. Climate change, firm performance, and investor surprises. Management Science. 2023; 69: 7352–7398.

[27] Zhang P, Deschenes O, Meng K, et al. Temperature effects on productivity and factor reallocation: Evidence from a half million Chinese manufacturing plants. Journal of Environmental Economics and Management. 2018; 88: 1–17.

[28] Cai X, Lu Y, Wang J. The impact of temperature on manufacturing worker productivity: evidence from personnel data. Journal of Comparative Economics. 2018; 46(4): 889–905.

[29] Zander K K, Botzen W J W, Oppermann E, et al. Heat Stress Causes Substantial Labour Productivity Loss in Australia. Nature Climate Change. 2015; 5(7): 647–651.

[30] Somanathan E, Somanathan R, Sudarshan A, Tewari M. The impact of temperature on productivity and labor supply: Evidence from Indian manufacturing. Journal of Political Economy. 2021; 129: 1797–1827.

[31] Karlsson J. Temperature and exports: evidence from the United States. Environmental and Resource Economics. 2021; 80(2): 311–337.

[32] Li C, Cong J, Yin L. Extreme heat and exports: Evidence from Chinese exporters. China Economic Review. 2021; 66: 101593.

[33] Zhang J, Li H. Will temperature affect the export quality of firms? Evidence from China. International Journal of Climate Change Strategies and Management. 2023; 15(4): 493–514.

[34] Vandenbussche H, Viegelahn C. Input reallocation within multi-product firms. Journal of International Economics. 2018; 114: 63–79.

[35] Deschênes O, Greenstone M. The economic impacts of climate change: evidence from agricultural output and random fluctuations in weather. American Economic Review. 2007; 97(1): 354–385.

[36] Cameron A Colin, Jonah B Gelbach, and Douglas L Miller. Robust inference with multiway clustering. Journal of Business & Economic Statistics. 2011; 29 (2).

[37] Dell M, Jones B F, Olken B A. What do we learn from the weather? The new climate-economy literature. Journal of Economic Literature. 2014; 52(3): 740–98.

[38] Yu M. Processing trade, tariff reductions and firm productivity: Evidence from Chinese firms. The Economic Journal. 2015; 125(585): 943–988.

[39] Manova K, Yu Z. How firms export: Processing vs. ordinary trade with financial frictions. Journal of International Economics. 2016; 100: 120–137.

[40] Dai M, Maitra M, Yu M. Unexceptional exporter performance in China? The role of processing trade. Journal of Development Economics. 2016; 121: 177–189.

[41] Adhvaryu A, Kala N, Nyshadham A. The light and the heat: Productivity co-benefits of energy-saving technology. Review of Economics and Statistics. 2020; 102(4): 779–792.

[42] Peng Y, Zheng R, Yuan T, et al. Evaluating perception of community resilience to typhoon disasters in China based on grey relational TOPSIS model. International Journal of Disaster Risk Reduction. 2023; 84: 103468.

[43] Mata D D, Resende G. Changing the climate for banking: The economic effects of credit in a climate-vulnerable area. Journal of Development Economics. 2020; 146, 102459.

[44] Chan FKS, Chen WY, Gu XB, Peng Y, Sang YF. Transformation towards resilient sponge city in China. Nature Reviews Earth & Environment. 2022; 3(2), 99–101.

[45] Hausmann R, Hwang J, Rodrik D. What you export matters. Journal of Economic Growth. 2007; 12(1):1–25.

