# Correction: Climate effects on the diversification strategy of export firms: Evidence from China

**DOI:** 10.1371/journal.pone.0318385

**Published:** 2025-01-24

**Authors:** Junmei Zhang, Lianying Yao

There are errors in the Funding statement. The correct Funding statement is as follows: JZ is the recipient of Zhejiang Office of Philosophy and Social Science grant #24NDQN165YBM and Zhejiang Province Public Welfare Technology Application Research Project #LQ24G030007. LY received funding from the National Social Science Fund of China, grant #23BZZ012. This study is supported by the Zhejiang Provincial Social Science Planning. The funders played no role in study design, data collection and analysis, publication decisions, or manuscript preparation.
